# *Phytophthora heterospora* sp. nov., a New Pseudoconidia-Producing Sister Species of *P. palmivora*

**DOI:** 10.3390/jof7100870

**Published:** 2021-10-16

**Authors:** Bruno Scanu, Thomas Jung, Hossein Masigol, Benedetto Teodoro Linaldeddu, Marília Horta Jung, Andrea Brandano, Reza Mostowfizadeh-Ghalamfarsa, Josef Janoušek, Mario Riolo, Santa Olga Cacciola

**Affiliations:** 1 Department of Agricultural Sciences, University of Sassari, 07100 Sassari, Italy; abrandano@uniss.it; 2 Phytophthora Research Centre, Faculty of Forestry and Wood Technology, Mendel University in Brno, 613 00 Brno, Czech Republic; thomas.jung@mendelu.cz (T.J.); marilia.jung@mendelu.cz (M.H.J.); josef.janousek@mendelu.cz (J.J.); 3 Phytophthora Research and Consultancy, 83131 Nußdorf, Germany; 4 Department of Plant Protection, School of Agriculture, Shiraz University, Shiraz 7144165186, Iran; hossein.masigol@gmail.com (H.M.); rmostofi@shirazu.ac.ir (R.M.-G.); 5 Dipartimento Territorio e Sistemi Agro-Forestali, Università Degli Studi di Padova, 35020 Legnaro, Italy; benedetto.linaldeddu@unipd.it; 6 Department of Agricultural Science, Mediterranean University of Reggio Calabria, 89122 Reggio Calabria, Italy; mario.riolo@unirc.it; 7 Department of Agriculture, Food and Environment, University of Catania, 95123 Catania, Italy; olga.cacciola@unict.it

**Keywords:** oomycete, Peronosporaceae, *Phytophthora*, clade 4, taxonomy, pseudoconidia, sporangia, multi gene sequencing, phylogenetic analyses, olive, durian

## Abstract

Since 1999, an unusual *Phytophthora* species has repeatedly been found associated with stem lesions and root and collar rot on young olive trees in Southern Italy. In all cases, this species was obtained from recently established commercial plantations or from nursery plants. Morphologically, the *Phytophthora* isolates were characterized by the abundant production of caducous non-papillate conidia-like sporangia (pseudoconidia) and caducous papillate sporangia with a short pedicel, resembling *P. palmivora* var. *heterocystica*. Additional isolates with similar features were obtained from nursery plants of *Ziziphus spina-christi* in Iran, *Juniperus oxycedrus* and *Capparis spinosa* in Italy, and mature trees in commercial farms of *Durio zibethinus* in Vietnam. In this study, morphology, breeding system and growth characteristics of these *Phytophthora* isolates with peculiar features were examined, and combined mitochondrial and nuclear multigene phylogenetic analyses were performed. The proportion between pseudoconidia and sporangia varied amongst isolates and depended on the availability of free water. Oogonia with amphigynous antheridia and aplerotic oospores were produced in dual cultures with an A2 mating type strain of *P. palmivora*, indicating all isolates were A1 mating type. Phylogenetically, these isolates grouped in a distinct well-supported clade sister to *P. palmivora*; thus, they constitute a separate taxon. The new species, described here as *Phytophthora heterospora* sp. nov., proved to be highly pathogenic to both olive and durian plants in stem inoculation tests.

## 1. Introduction

The genus *Phytophthora* de Bary (Peronosporaceae, Peronosporales, kingdom Stramenipila) is one of the most important groups of plant pathogens, causing a range of diseases in agricultural, horticultural, forest, and natural ecosystems worldwide [1,2,3]. Initially, the taxonomic description of *Phytophthora* species was exclusively based on morphological analyses of reproductive structures, such as sporangia, gametangia, chlamydospores, and hyphal swellings, as well as colony morphology and growth rate at different temperatures [1]. With the advent of molecular DNA techniques and the development of phylogenetic inference, our understanding of the systematic and diversity of *Phytophthora* species has changed considerably over time [3,4,5]. Currently almost 200 species are officially described, grouped into 12 distinct and well supported phylogenetic clades and numerous sub-clades [4,6]. The majority of the described *Phytophthora* species are soilborne and waterborne, primarily responsible for root and collar rots, and occasionally bleeding stem cankers on several plant hosts [1,3,5,7]. These species are characterized by the production of persistent sporangia, and their infection occurs through the release of biflagellate zoospores into soil or surface water, which are then attracted by chemical or electrical signals generated by the plant host [8]. Airborne *Phytophthora* species, on the other hand, produce almost exclusively caducous sporangia and primarily infect aerial parts of plants, causing leaf necroses, shoot blights, fruit rots, and bleeding bark cankers on stem and branches [1,3,5]. In this case, infections occur through detached sporangia spread by wind and rain splash that can either germinate directly to produce mycelia (which in turn can differentiate further sporangia) or indirectly by releasing zoospores [3,7,9]. Finally, there are some *Phytophthora* species with a mixed epidemiological strategy, having both caducous and persistent sporangia, thereby behaving as both soil- and airborne pathogens [3,5,7,10].

**Figure 1:**
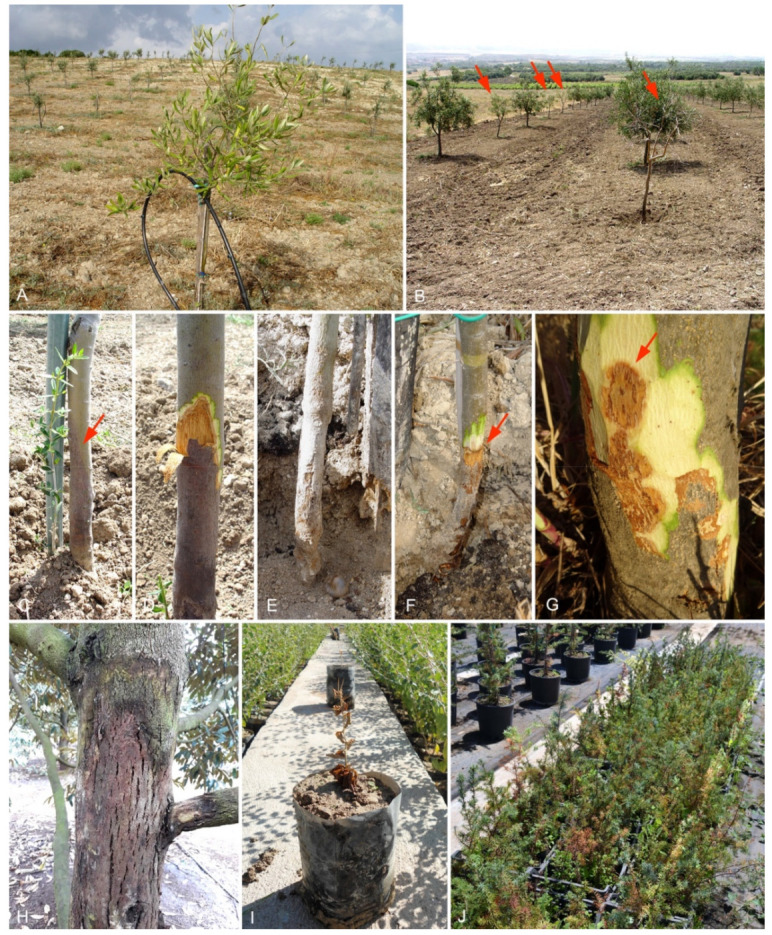
Disease symptoms caused by *Phytophthora heterospora* on *Olea europaea* in Italy (**A**–**G**): (**A**) 3- to 4-year-old trees showing chlorosis and increased transparency of the crown; (**B**) a 5-year-old plantation with dying and dead trees; (**C**) necrotic outer bark lesion on the lower part of the stem (arrow); (**D**) orange-brown and flame shaped lesions on the stem; (**E**,**F**) necrotic bark lesions girdling the collar; (**G**) single-spot bark lesions on the main stem (arrow). (**H**) Stem canker and scaffold branches on a mature tree of *Durio zibethinus* in Vietnam. (**I**) A dead 1-year-old seedling of *Ziziphus spina-christi* in a nursery in Iran. (**J**) Two-year-old *Juniperus oxycedrus* seedlings showing dieback in a nursery in Italy.

In 2010, a new disease caused by *Phytophthora* was reported on 3- to 4-year-old olive (*Olea europaea* L. cv. Bosana) trees in recently established plantations and commercial nurseries in Sardinia, Italy [11]. Specific symptoms consisted of leaf chlorosis, wilting, defoliation, and dieback, eventually followed by plant death (Figure 1A,B). This syndrome was associated with orange-brown and flame shaped necrosis in the inner bark, originating from root or collar infections and developing up to 50 cm in the stem (Figure 1C–F). Single necrotic spots, unconnected to collar lesions, often occurred along stems (Figure 1G), indicating that bark necrosis might also have originated from aerial infections. The disease resulted in severe dieback and mortality of olive trees, with an incidence ranging from 20% to 60%. Isolations from infected bark tissues consistently yielded a *Phytophthora* species that, based on morphological characters and 99% similarity of the internal transcribed spacer (ITS) sequences, was initially identified as *Phytophthora palmivora* (E.J. Butler) E.J. Butler [11]. Most of the isolates, however, behaved unusually—in addition to producing the typical papillate sporangia of *P. palmivora*, the isolates also produced conidia-like sporangia that exclusively germinated directly via germ tubes. This unique feature was already previously reported from a few isolates obtained from root rot of young olive trees in Calabria, Italy, also identified as *P. palmivora*, although no DNA sequences were generated at that time [12,13]. Due to their resemblance to the conidia of haploid fungi, the conidia-like sporangia of this unusual diploid *Phytophthora* species are in the following named as pseudoconidia. Similar symptoms to those described on olive in Italy were detected in 2013 in the Mekong River delta (Vietnam) on mature, fruit-bearing trees of durian (*Durio zibethinus* L.), one of the most appreciated and profitable fruit crops in this country. Durian trees showed aerial stem cankers with gum exudates and longitudinal cracking of the bark (Figure 1H), from which a *Phytophthora* species was consistently isolated and morphologically identified as *P. palmivora*. Additional isolates, with identical ITS sequences to those of the isolates recovered from olive trees, were detected from the crown and root rot of *Ziziphus spina-christi* (L.) Desf. nursery seedlings in Kazerun County (Fars Province, Iran) in 2011 and from *Juniperus oxycedrus* L. and *Capparis spinosa* L. nursery plants in Italy in 2013 and 2014, respectively.

In all cases, the isolates were characterized by the production of pseudoconidia that resembled the sporocysts described for *P. palmivora* var. *heterocystica* Babacauh from *Theobroma cacao* L. in the Ivory Coast in 1983 [1,14], for which, unfortunately, no specimens or DNA sequence data are available. Therefore, in this study, extensive morphological, physiological, and phylogenetic analyses of the unusual *Phytophthora* isolates from Italy, Iran, and Vietnam were performed, and comparisons were made to *P. palmivora*, resulting in the description of *Phytophthora heterospora* sp. nov.

## 2. Materials and Methods

### 2.1. Phytophthora Isolation and Culture Maintenance

From olive trees *Phytophthora* isolations were mostly made from necrotic bark lesions on stems, collars, and roots. Small pieces from the margins of fresh lesions were cut aseptically and plated onto synthetic mucor agar (SMA) [15]. Soil and fine roots of infected trees were also collected and baited with *Rhododendron* sp. and citrus leaves using the method originally described by Jung et al. [16]. From *C. spinosa*, *D. zibethinus*, *J. oxycedrus*, and *Z. spina-christi* seedlings, *Phytophthora* isolates were obtained by both direct isolation from stem lesions, infected roots, and collar tissues and baiting of the rhizosphere soil using fresh cork oak and citrus leaves as baits and selective SMA and CMA-PARP agar [17]. Any colonies developing from infected tissues and leaf baits on selective media were subcultured onto carrot agar (CA; Oxoid^®^ n°3 agar 16 g L^−1^, CaCO_3_ 3 g L^−1^, blended carrots 200 g L^−1^) and cornmeal agar (CMA; Oxoid^®^ ground corn extract 40 g L^−1^, agar 15 g L^−1^), incubated at 20 °C and examined within 4 days using morphological characters for identification [15].

Six isolates of *P. palmivora*, including the representative specimen type “S” (CBS 179.26) proposed by Brasier and Griffin [18], were included for interspecific comparisons (Table 1 and Table S1). Stock cultures were maintained on CA slopes at 15 °C in the dark and preserved in the culture collections of the University of Sassari, Italy, and the University of Catania, Italy. Dried culture holotypes were lodged with the CBS Herbarium, ex-type and paratype cultures were deposited at the Westerdijk Fungal Biodiversity Institute (CBS; Utrecht, The Netherlands), and the novel taxonomic description and nomenclature were submitted to MycoBank (www.mycobank.org).

### 2.2. DNA Extraction, Amplification, and Sequencing

Extraction of mycelial DNA of the *P. heterospora* and *P. palmivora* isolates used in the morphological studies was performed using the InstaGene Matrix (Bio-Rad Laboratories, Hercules, CA, USA) and the DNGTM-PLUS (Cinnagen, Tehran, Iran) kits following the manufacturer’s instructions. The resulting DNA was stored at −20 °C, and its quantity and quality were determined using a MD-100 Nanodrop machine (NanoDrop Technologies, Wilmington, DE, USA). The Internal Transcribed Spacers of the ribosomal RNA (ITS) were amplified and sequenced using primers ITS-6 [22] and ITS-4 [23]. Additional gene regions were amplified and sequenced; (1) β-tubulin (*Btub*) was amplified using primers Btub F1 and Btub R1 [24], (2) the mitochondrial genes cytochrome c oxidase subunit 1 (*cox*1) was amplified with primers FM83 and FM84 [25], and (3) NADH dehydrogenase subunit 1 (*nadh*1) was amplified with NADHF1 and NADHR1 primer [26]. PCR conditions and reaction mixture were as described previously [24,26], except for the amplification conditions for the *cox*1 that consisted of 1 cycle of 95 °C for 2 min, followed by 35 cycles of 94 °C for 40 s, 55 °C for 50 s, 72 °C for 1 min, and a final extension step of 7 min at 72 °C. The PCR products were purified using the EUROGOLD gel extraction kit (EuroClone S.p.A., Italy) following manufacturer’s instructions. All gene regions were sequenced in both directions with primers used in amplification by the BMR Genomics DNA sequencing service (www.bmrgenomics.it). DNA sequence chromatograms were viewed and edited using BioEdit v. 5.0.6 software [27]. DNA isolation, amplification and sequencing of additional loci of oomycete isolates needed for phylogenetic analysis was performed as described previously [28]. Heterozygous sites observed were labelled according to the IUPAC coding system. All sequences derived in this study were submitted to GenBank (http://www.ncbi.nlm.nih.gov/) and accession numbers are given in Table S1.

### 2.3. Phylogenetic Analyses

For phylogenetic analyses, the sequences obtained in this study were complemented with publicly available sequences of *Phytophthora* isolates representative of all main phylogenetic clades (Table S1). Furthermore, loci of representative downy mildews, including grass-infecting graminicolous downy mildews (GDM), downy mildews with pyriform haustoria (DMPH), downy mildews with colored conidia (DMCC), and brassicolous downy mildews (BDM), were added to the sequence’s dataset (Table S1). The source databases were the GenBank Nucleotide Collection and GenBank Whole-Genome Shotgun contigs. In some cases, sequences from two isolates from the same species were combined into a single sample because neither of them had all desired loci available in GenBank (Table S1); this was only performed if the two isolates shared at least one identical sequence. *Nothophytophthora valdiviana* sequences were included as outgroup taxon.

The sequences of the loci used in the analyses were aligned using the online version of MAFFT v. 7 [29] by the E-INS-I strategy (ITS) or the G-INS-I strategy (all other loci). Many of the downy mildew species are known to have extremely long ITS sequences of up to more than 3000 bp, caused by long repetitive insertions, which can affect both ITS1 and ITS2 [30]. The ITS alignment in this study was manually edited and adjusted, and all insertions longer than 50 bp present exclusively in downy mildews isolates were removed (in total 5729 characters). To sort out the phylogenetic position of *P. heterospora* within phylogenetic clade 4, a concatenated 4-partition dataset of the nuclear (ITS, *Btub*) and mitochondrial loci (*cox*1, *nadh*1), consisting of 4161 characters, was analyzed.

Bayesian Inference (BI) analysis was performed using MrBayes version 3.2.7 [31,32] into partitions with the GTR Gamma + I nucleotide substitution model. Four Markov chains were run for 20 M generations, sampling every 1000 steps and with a burn in at 9000 trees. Maximum Likelihood (ML) analysis was carried out using the raxmlGUI v. 2.0 [33] implementation of RAxML [34] with a GTR Gamma + I nucleotide substitution model. There were 10 runs of the ML and bootstrap (thorough bootstrap) analyses with 1000 replicates used to test the support of the branches. Phylogenetic trees were visualized in Mesquite version 3.61 [35] and/or MEGA X version 10.2.6 [36] and were edited in figure editor programs. Datasets presented and original trees deriving from BI and ML were deposited at Dryad Dataset (https://datadryad.org).

### 2.4. Morphological Characterization and Cardinal Temperature for Growth

Ten isolates of *P. heterospora* and four isolates of *P. palmivora* were included in the morphological studies. Measurements and photos of morphological structures were made at 400× magnification and recorded using a digital camera Leica DFC495 connected to a Leitz Diaplan compound microscope (Leitz, Germany) and Leica Application Suite imaging software v.4.5.0 (Leica Microsystems, Switzerland). Pseudoconidia and sporangia were examined both on solid CA after 7 days at 20 °C and from CA plugs (10 mm diameter) placed in 60 mm Petri dishes flooded with nonsterile soil extract water. Chlamydospores and hyphal swellings were examined directly on CA plates, if present. Length (l), breadth (b), l/b ratio, pedicel length, and characteristic features of pseudoconidia and sporangia, as well as shape and diameters of chlamydospores and hyphal swellings of 50 mature structures randomly selected, were recorded for each isolate [37]. In addition, the proportion between pseudoconidia and sporangia was assessed across the isolates, both in water and on solid CA. Specifically, four 1 mm^2^ discs were taken from each *P. heterospora* isolate listed in Table 1 and mounted on glass slides with sterile water. The number of sporangia contained in each 1 mm^2^ was counted using a Leitz Diaplan compound microscope. The morphology of pseudoconidia was observed also through a scanning electron microscopy (SEM). CA plugs of 4 × 4 mm taken from 7-day-old cultures were fixed in 2% glutaraldehyde in 0.1 M sodium-cacodylate buffer (EMS), pH 7.2, for 1 h at 4 °C and then post-fixed in 1% osmium tetroxide (EMS) for 1 h at 4 °C. After dehydration in graded ethanol and critical point drying using CO_2_ (Emscope-CPD 750), the samples were attached by CCC carbon adhesive directly on the microscope stubs, coated with vacuum evaporated gold (Emscope-SM 300), and observed using a Field Emission Scanning Electron Microscope (FESEM).

The sexual compatibility type of all *P. heterospora* isolates was determined in dual culture with known A1 and A2 tester strains of *P. cinnamomi* (P904, P1889) on CA [15]. Selfed gametangia of the six representative isolates were induced on 35 mm plates containing 10 mL CA in polycarbonate membrane tests (Whatman Nuclepore™ Track-Etched Membranes, Sigma-Aldrich, St. Louis, MO, USA) with opposite mating type tester strains of *P. palmivora* (CBS 179.26). Paired cultures were incubated at 20 °C in darkness and scored for gametangial formation after 15–20 days. Fifty gametangia were chosen at random, and dimensions and characteristic features of antheridia, oogonia, and oospores were measured and recorded at 400× magnification. The oospore aplerotic index and wall index were calculated according to Dick [38].

Colony morphologies were characterized from 4-day-old cultures incubated at 20 °C in the dark on CA, V8-juice agar (V8A; filtered V8 juice 100 mL L^−1^, CaCO_3_ 0.1 g L^−1^) [15], potato dextrose agar (PDA; 39 g L^−1^), and malt extract agar (MEA; 16 g L^−1^) (all agar media were sourced from Oxoid^®^, Basingstoke, UK). Temperature–growth rate studies were performed on CA according to Scanu et al. [15]. Each isolate was incubated with three replicates at 5, 10, 15, 20, 25, 27.5, 30, 32.5, 35, and 40 °C (all ±0.5 °C).

### 2.5. Pathogenicity Test

To fulfill Koch’s postulates, the pathogenicity of three representative isolates of *P. heterospora* was tested against 2-year-old olive (*Olea europaea* cv. Bosana) saplings. One isolate of *P. palmivora* and one isolate of *P.* taxon *palmivora*-like were included in the trial. The experimental design consisted of five replicates (saplings) per isolate, and five saplings, inoculated with a CA plug, were used as negative controls. A 5 mm diameter hole through the bark of the sapwood stem was made using a cork borer. Then, a 5 mm agar plug taken from the margin of 4-day-old cultures grown on CA was inserted into the wound. Soon after inoculation, wounds were wrapped with sterile damp cotton wool and covered with aluminum foil and parafilm. Saplings were maintained in the laboratory for 2 months in daylight and at temperatures ranging between 25 and 30 °C. At the end of the experiment, the outer bark was stripped and the necrotic lesion area surrounding each inoculation point was traced and then measured using APS Assess 2.0 software (APS Press) [10]. Re-isolation was attempted by transferring ten pieces taken from the margin of each lesion to SMA and incubating the plates at 20 °C.

The pathogenicity of the *P. heterospora* isolate DB2 was tested on 12-year-old durian trees in a commercial farm in the Dong Nai province (southern Vietnam). Inoculation was performed in December 2013 by underbark-inoculation. A colony plug (5 mm diam) was inserted under the bark of branches of the trees (two trees and three branches for each tree). The bark disk was put back in place to plug the wound, which was covered with a sterile moist cotton plug and sealed with adhesive tape. Branches inoculated with sterile agar served as a control. During the experiment, temperature ranged between 25 °C (night) and 32.5 °C (day). Natural light with around 12 h of light. Symptoms were first recorded 15 days after inoculation. For measuring the size of the necrotic lesions, the bark was removed 30 days after inoculation.

## 3. Results

### 3.1. Phylogeny

The aligned datasets for the nuclear (ITS, *Btub*) and mitochondrial (*cox*1, *nadh*1) genes consisted of 1101, 918, 1346, and 796 characters, respectively. Across the concatenated 4141 characters alignment of all four gene regions, *P. heterospora* showed 21–28 unique polymorphisms (Table S2). All isolates of *P. heterospora* were identical across all four loci apart from positions 307 and 438 in *Btub*, which were heterozygous in several isolates, and position 660 in *Btub*, where isolate PH211 had an A instead of a G. The mitochondrial sequences of the two isolates PH083 and PH090 from *A. unedo*, informally designated here as *P.* taxon *palmivora*-like, were identical to those of *P. heterospora*.

**Figure 2:**
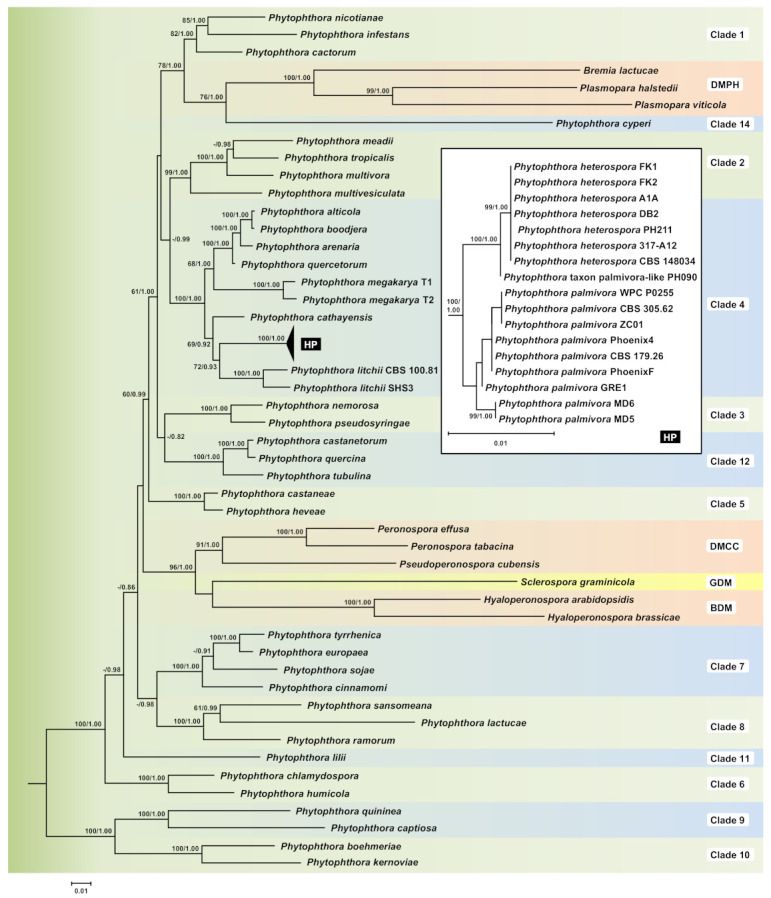
Fifty percent majority rule consensus phylogram derived from maximum likelihood analysis of a concatenated four-locus (ITS, *Btub*, *cox*1, *nadh*1) dataset of representative species from phylogenetic clades 1–12 and 14 of *Phytophthora* and the four downy mildew groups DMPH, DMCC, GDM, and BDM. Maximum likelihood bootstrap values and Bayesian posterior probabilities are indicated but not shown below 60% and 0.80, respectively. *Nothophytophthora valdiviana* was used as outgroup taxon (not shown). Scale bar = 0.01 expected changes per site per branch.

To resolve the phylogenetic positions of *P. heterospora* within the genus *Phytophthora* and its separation from *P. palmivora*, a concatenated nuclear and mitochondrial 4-partition dataset (4141 characters) was analyzed. Support for terminal clades and their clustering was similar in both BI and ML analyses, and the ML analysis is presented here with both ML bootstrap and BI posterior probability values included (Figure 2, Dryad Dataset, https://doi.org/10.5061/dryad.1jwstqjvx). The isolates of *P. heterospora* formed a fully supported monophyletic cluster (BI posterior probability = 1.00, ML bootstrap = 99%), which resided in clade 4 in a close position to *P. palmivora*. Interestingly, isolate PH090 clustered in a strongly supported basal position to *P. heterospora*, intermediate between the latter and *P. palmivora* and, hence, is designated here as *P.* taxon *palmivora*-like (Figure 2). Amongst the isolates of *P. palmivora*, there was considerably higher intraspecific variability than in *P. heterospora*, with the two isolates from *Artocarpus heterophyllus* (MD5 and MD6) forming a distinct lineage (Figure 2). The phylogenetic analyses of the 65-taxon dataset, which included *Phytophthora*, *Nothophytophthora*, and representatives of all downy mildew groups, placed the latter into two fully supported monophyletic clades nested within the genus *Phytophthora*. The DMCC, GDM, and BDM clade resided in sister position to a large cluster comprising *Phytophthora* clades 1–5, 12, and 14 and the DMPH, whereas the latter clustered together with the obligate biotrophic *Phytophthora cyperi* from clade 14 in a sister position to clade 1 (Figure 2).

### 3.2. Taxonomy

**Figure 3:**
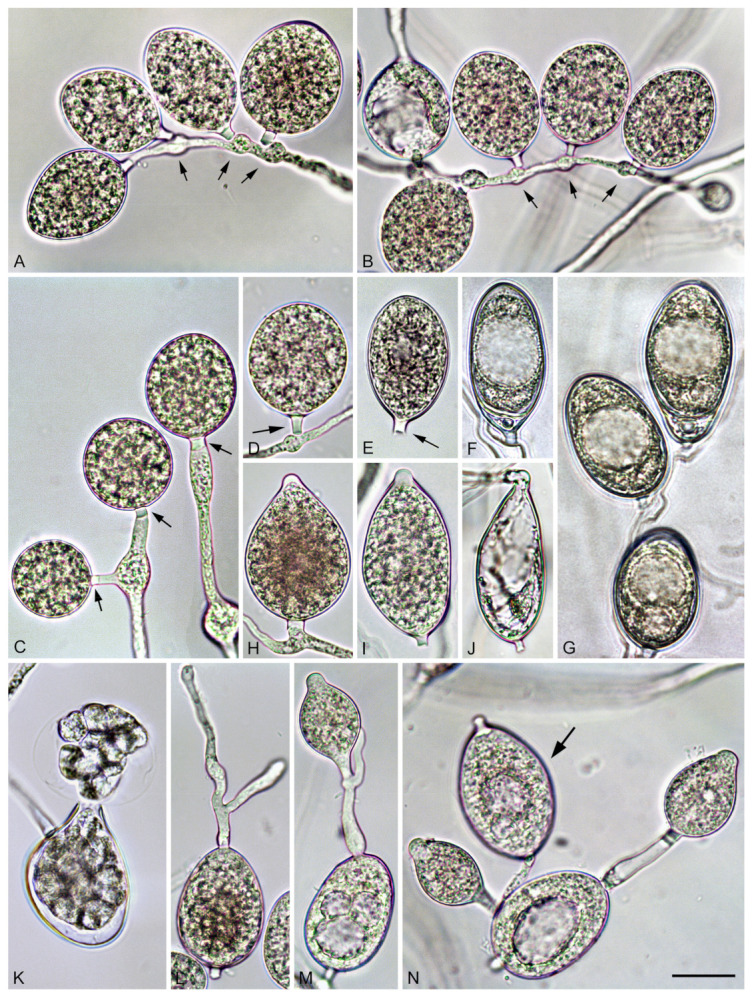
Pseudoconidia and sporangia of *Phytophthora heterospora*. (**A**,**B**) Ovoid pseudoconidia forming monochasial helicoid sympodia, still attached to the conidiophores or recently detached with nodose swellings (arrows) at the insertion points of the pseudoconidia to the conidiophores; (**C**) spherical to ovoid pseudoconidia with short pre-formed pedicels (arrows); (**D**,**E**) detached pseudoconidia with short pedicels (arrows); (**F**) ellipsoid conidium, with large lipid globule, tapering base, and external proliferation; (**G**) catenulate ellipsoid pseudoconidia with large lipid globules, forming a monochasial sympodium; (**H**) ovoid, laterally attached papillate sporangium; (**I**) limoniform papillate sporangium with short pedicel; (**J**) empty sporangium with direct germination through the apex; (**K**) sporangium releasing zoospores; (**L**–**N**) pseudoconidia germinating by forming papillate microsporangia and a new conidium (arrow). Scale bar: 25 µm.

**Figure 4:**
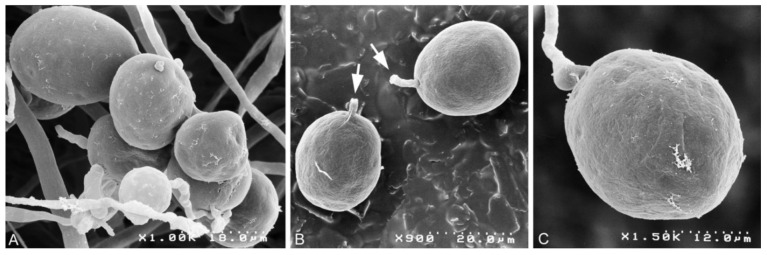
Scanning electron microscopy (SEM) images of pseudoconidia formed by *Phytophthora heterospora* on carrot agar: (**A**) cluster of pseudoconidia; (**B**) detached globose pseudoconidia with short pedicels (arrows); (**C**) ovoid pseudoconidium still attached to the conidiophore. Magnifications and scale bar dimensions are given in the right-bottom corners.

**Figure 5:**
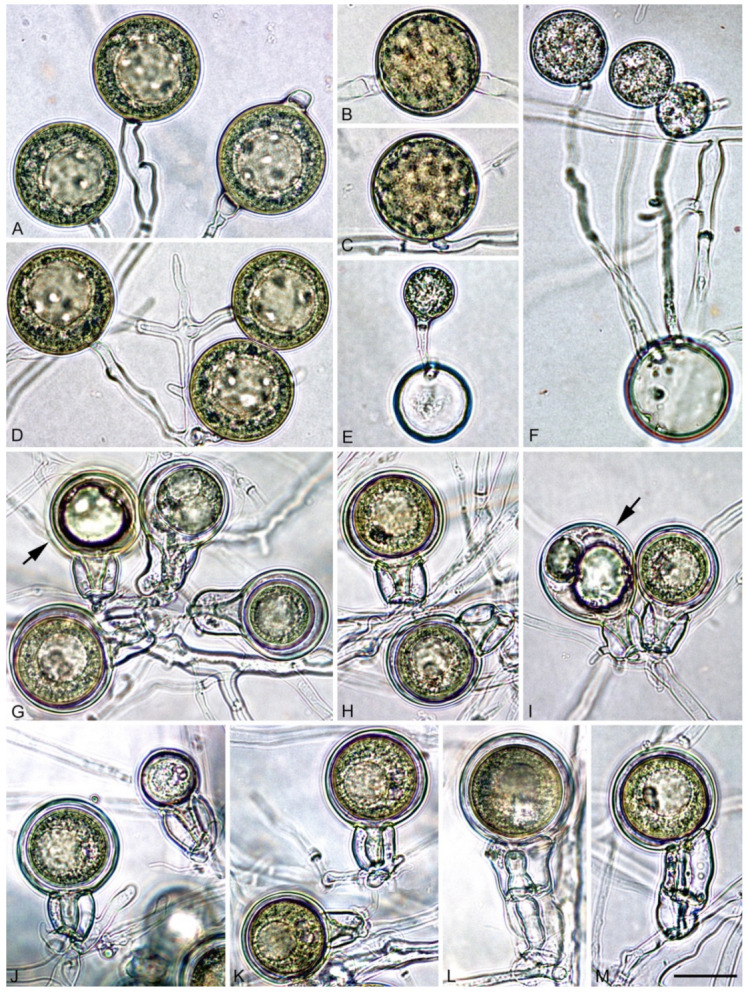
Chlamydospores and gametangia of *Phytophthora heterospora*. (**A**–**F**) Thick walled, globose chlamydospores; (**A**,**D**) terminal with lipid globules; (**B**) intercalary; (**C**) laterally attached chlamydospores; (**E**,**F**) germinating chlamydospores with one or more germ tubes forming new chlamydospores; (**G**–**M**) globose oogonia with aplerotic oospores containing large lipid globules and amphigynous antheridia; (**G**) oogonia with short tapering bases formed in a clump, one with aborted oospores (arrow); (**I**) oogonia with short tapering bases, one with aborted oospore and finger-like projection on the antheridium (arrow); (**J**,**K**) oogonia with short unicellular antheridia; (**L**) oogonia with very long bicellular antheridium; (**M**) comma-shaped oogonium with long bicellular antheridium. Scale bar: 25 µm.

*Phytophthora heterospora* Scanu, Cacciola, Linaldeddu & Jung sp. nov. (Figure 3, Figure 4 and Figure 5).

MycoBank MB 841284.

Etymology: the name refers to the production of a variety of different spores including pseudoconidia, zoospore-releasing sporangia, chlamydospores, and oospores.

Holotype: Italy, Sardinia, Sorso; isolated from a bark lesion on a young olive tree. Collected: B. Scanu, 2010; CBS H-24777 (holotype, dried culture on CA, Herbarium CBS-KNAW Fungal Biodiversity Centre), CBS 148034 = PH054 (ex-holotype culture). ITS, *Btub*, *cox*1 and *nadh*1 sequences GenBank MT232393, MZ782807, MZ782828, MZ782849, respectively.

Description: pseudoconidia were abundantly produced in solid media (CA and V8A) but less frequently formed in non-sterile soil extract water (Figure 3A–G). Shapes of pseudoconidia showed a wide variation ranging from ellipsoid (over all isolates 76.4%; Figure 3A,B,E–G) or subglobose to globose (14.8%; Figure 3C,D) or ovoid (6.8%; Figure 3C,D) and limoniform (2.0%), sometimes containing a large vacuole at maturity (Figure 3F,G). Pseudoconidia had mostly round or less frequently tapered bases, proliferating externally with hyphae arising close to the base and mainly forming monochasial helicoid sympodia (Figure 3A–C). Empty pseudoconidia were characterized by a thick wall. All pseudoconidia were caducous, showing a short pre-formed pedicel < 5 µm (av. 3.9 ± 0.4 µm), which breaks off from the bearing conidiophore (Figure 4B). Nodose swellings were common at the insertion points of the pseudoconidia to the conidiophore (Figure 3A–D). The pseudoconidia dimensions of the 10 isolates of *P. heterospora* averaged 34.7 ± 1.8 µm in length (l) and 27.1 ± 1.1 µm in breadth (b) (overall range 24.0–44.2 × 16.5–27.1 µm) with a l/b ratio of 1.6 ± 0.1 (range of isolate means 1.3–1.7). Pseudoconidia did not release zoospores but germinated directly with one or more germ tubes, which usually emerged through the conidial wall and originated new pseudoconidia or papillate microsporangia (Figure 3L–N).

Sporangia of *P. heterospora* were readily produced in non-sterile soil extract water but were more rarely observed in solid agar (CA and V8A) (Figure 3H–J,L–N), typically borne terminally on unbranched sporangiophores or in irregular lax or regular dense sympodia. Sporangia often originated from a conidium (Figure 3L–N) and proliferated externally (Figure 3M). Sporangial shapes were diverse ranging from ovoid (overall isolates 68.4%; Figure 3H,N) or elongate-ovoid to elongate-obpyriform (14.8%; Figure 3J) or limoniform (6.8%; Figure 3I,L–N), mouse to distorted shapes (6.3%) and subglobose (3.7%), often with laterally attached sporangiophore (48.6%), occasionally with markedly curved apex (8.6%) and presence of a vacuole (8.0%; Figure 3I,N) and rarely with a hyphal appendix. Sporangia were caducous, with a short pedicel < 5 µm (av. 3.6 ± 0.5 µm), papillate or very rarely bipapillate (overall isolates < 1%). The sporangial dimensions averaged 42.8 ± 5.9 µm in length (l) and 24.7 ± 3.5 µm in breadth (b) (overall range 15.6–72.6 × 12.4–42.0 µm) with a l/b ratio of 1.7 ± 0.1 (range of isolate means 1.4–1.9). Sporangia germinated directly by forming a germ tube (Figure 3J) that originated a new sporangium or indirectly by releasing zoospores (Figure 3K). Zoospores limoniform to reniform whilst motile, becoming spherical (av. diam = 8.8 ± 1.2 µm) on encystment. The proportion of pseudoconidia vs. sporangia varied based on the isolate and cultural conditions, with two major groups behaving differently (Table 2).

Hyphal swellings were frequently occurring along the sporangiophores and conidiophores, subglobose to mostly globose, intercalary, catenulate averaging 10.4 ± 3.6 µm. Diameters of primary hyphae ranged from 3.2 to 6.8 µm. Chlamydospores were abundantly produced on CA and V8A (Figure 5A–F); they were thick-walled, globose, and terminally (Figure 5A,D), intercalary (Figure 5B), or laterally (Figure 5C) inserted, with 28.4 ± 4.4 µm diameter (overall range 19.3–43.0 µm). Chlamydospores often germinated with one or more germ tubes originating new smaller chlamydospores (Figure 5E,F).

All *P. heterospora* isolates were self-sterile and produced gametangia readily when paired with A2 isolates of *P. palmivora* using the polycarbonate membrane test on CA (Figure 5K–N). No gametangia were produced when isolates of *P. heterospora* were paired with each other or with A1 mating-type tester strains of *P. cinnamomi*. Oogonia were globose, sometimes slightly comma-shaped (Figure 5M), often with short tapering bases (av. 74.6%; Figure 5G–K), or elongated with long and often distorted bases (av. 25.4%; Figure 5L,M), produced in pairs or clumps (Figure 5G–I) or single and borne laterally (Figure 5J–M). Oogonial walls were always smooth and occasionally turned golden-brown within 4 weeks (Figure 5G,H). Oogonial diameters averaged 30.5 ± 2.2 μm, with a total range of 24.3–37.4 μm. Oospores were aplerotic (av. 94.7%; Figure 5G,L,M) or infrequently plerotic (av. 5.3%; Figure 5H–K), contained large ooplasts, occasionally excentric, with thin walls (on av. 1.6 ± 0.3 μm) and a mean oospore wall index of 0.33 ± 0.06. Oospore abortion rates averaged 60% (Figure 5G,I). Antheridia were exclusively amphigynous, 51.4% unicellular (Figure 5G–K), and 48.6% bicellular (Figure 5L,M), averaging 15.3 ± 2.8 µm in length and 13.8 ± 1.5 µm in breadth, with a l/b ratio of 1.1 ± 0.1, often with one or more finger-like projections.

**Figure 6:**
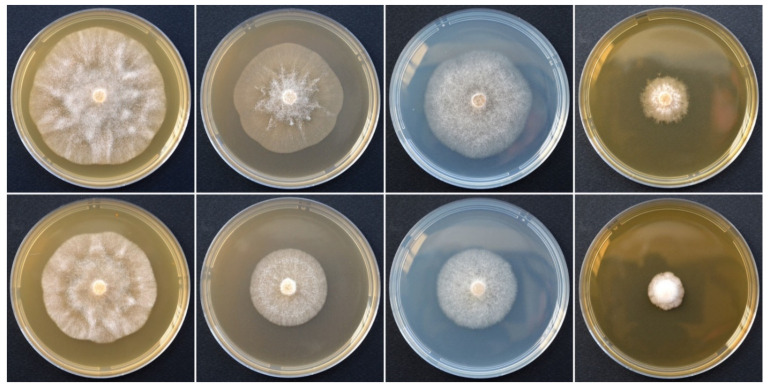
Colony morphologies of *Phytophthora heterospora* (upper row; isolate PH054) and *P. palmivora* (bottom row; isolate CBS 179.26) after 4 days growth at 20 °C in the dark on CA, V8A, PDA, and MEA (from left to right).

**Figure 7:**
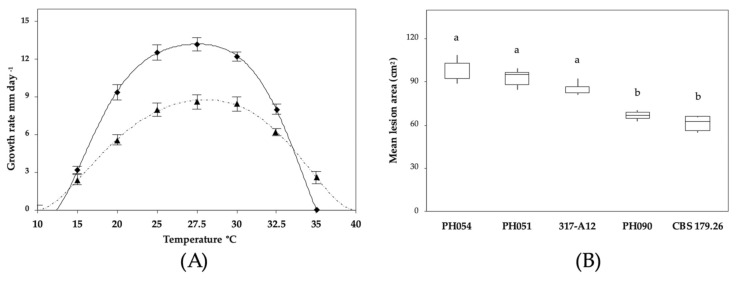
Temperature–growth relations and pathogenicity of *Phytophthora* taxa: (**A**) radial growth rates of *P. heterospora* (continuous line; 10 isolates) and *P. palmivora* (dashed line; 4 isolates) on CA at different temperatures. The data are plotted as average ± SD; (**B**) box and whiskers diagram showing mean lesion areas produced by three isolates of *P. heterospora* (PH054, PH051, and 317-A12), *P.* taxon *palmivora*-like (PH090), and *P. palmivora* (CBS 179.26) on 2-year-old olive saplings after 2 months. Control saplings did not show any necrotic lesions around the inoculation points and were not included. Different letters above bars indicate significant differences according to Fisher’s LSD test (*p* = 0.05).

Colony morphology and growth rates: *P. heterospora* colonies were radiate with limited aerial mycelium on CA and V8A, uniform and slightly woolly on PDA and poorly developed, dense-felty with irregular margin on MEA (Figure 6). Temperature–growth relations are shown in Figure 7A. All eight isolates included in the growth test had similar growth rates. The maximum growth temperature was around 32.5 °C, while the minimum temperature was above 10 °C. The average radial growth rate at the optimum temperature of 27.5 °C was 12.8 mm/d (Figure 7A).

Other specimens examined (paratypes): Italy, Sardinia, Villamar. Isolated from a collar lesion of a young *Olea europaea* tree. Collected by: B. Scanu, 2010; CBS H-24778, CBS 148035 = PH051. Sardinia, Villamar. Isolated from rhizosphere soil of *Olea europaea* saplings. Collected by: B. Scanu, 2010; PH052; PH057. Sardinia, Sorso. Isolated from rhizosphere soil of a young *Olea europaea* trees. Collected by: B. Scanu, 2010; PH047. Italy, Sicily, Catania. Isolated from *Capparis spinosa*. Collected by: S.O. Cacciola, 2014; CBS H-24779, CBS 148036 = 317-A12. Italy, Calabria, Lamezia Terme. Isolated from *Olea europaea*. Collected by: S.O. Cacciola, 1999; Palm2. Italy, Sicily, Campobello di Mazara. Isolated from *Olea europea*. Collected by: S.O. Cacciola, 2005; Campobello 2b. Vietnam, Mekong Delta, Vinh Long. Isolated from necrotic bark lesions on *Durio zibethinus* trees. Collected by: S.O. Cacciola, 2013; DB2; A1A; A1B1; C2B1.

Host/distribution: *Olea europaea*, *Juniperus oxycedrus*, and *Capparis spinosa* (Italy); *Ziziphus spina-christi* (Iran) and *Durio zibethinus* (Vietnam).

Notes: *Phytophthora heterospora* and *P. palmivora* are very similar in terms of colony morphology and sporangia, chlamydospores, and gametangia characteristics. However, *P. heterospora* can be easily distinguished by the production of pseudoconidia on solid agar media. Other key differences between *P. heterospora* and *P. palmivora* are (i) the lower maximum temperature for growth and faster growth rates at most temperatures of *P. heterospora*, and (ii) the higher oospore abortion rate of *P. heterospora* (60% vs. 48%). Phylogenetically, *P. heterospora* differs from *P. palmivora* in ITS, *Btub*, *cox*1, and *nadh*1 by 3–4, 11–16, 1–2, and 6 fixed polymorphisms, respectively. Although *P. heterospora* is present on four continents, its geographic distribution presently seems to be restricted to a few countries and mainly to nursery plants and new plantations, contrasting with the global distribution of *P. palmivora* in a wide range of natural, horticultural, and ornamental ecosystems and nurseries [1,18,39].

### 3.3. Pathogenicity

In the inoculation tests, both *P. heterospora* and *P. palmivora* were shown to be pathogenic to *O. europaea* (Figure 7B). All isolates used in the experiments were able to cause necrotic lesions significantly larger (*p* < 0.0001) than those of the negative controls. Overall, with mean lesion areas of 92.4 ± 8.2, *P. heterospora* was significantly (*p* < 0.05) more aggressive than *P. palmivora*, with 64.1 ± 5.2 cm^2^. The isolate PH054 was the most aggressive among the *P. heterospora* isolates, causing a mean lesion area of 98.2 ± 8.2, but intraspecific differences were statistically not significant (*p* > 0.05). Saplings inoculated with *Phytophthora* species developed the typical symptoms observed in the field, such as leaf chlorosis, wilting, and defoliation. *Phytophthora* taxon *palmivora*-like (PH090) were also pathogenic with necrotic lesions comparable with *P. palmivora*. All *Phytophthora* taxa were re-isolated from necrotic stem lesions.

On durian branches wound-inoculated with the Vietnamese *P. heterospora* isolate DB2 from durian, the first symptoms, consisting of gum exudation and bark necrosis, appeared 15 days after inoculation. After 30 days, the mean area of necrotic lesions in inoculated branches was 96 ± 10 mm^2^. No symptoms were observed on the control branches.

## 4. Discussion

Species in the genus *Phytophthora* are characterized by the production of asexual sporangia releasing biflagellate zoospores into an evanescent vesicle that soon breaks, allowing zoospores to swim into the water [1]. The occurrence of an additional asexual dissemination structure in *P. heterospora*, designated here as pseudoconidium, which germinates directly instead of releasing zoospores, represents a unique feature of the genus *Phytophthora*. This trait, referred to as sporocyst, was previously reported by Babacauh in 1983 on isolates obtained from *T. cacao* in the Ivory Coast, designated as *P. palmivora* var. *heterocystica* because of the differentiation of unusual conidia-like sporangia [1,14]. Since no specimens or DNA sequence data linked to the original description are available and no further reports of this variety have been published, it remains unclear whether *P. heterospora* and *P. palmivora* var. *heterocystica* belong to the same taxon, although it cannot be ruled out.

As stated by Babacauh [1,14], the occurrence of pseudoconidia may be an evolutionary trend towards less dependence on free water, as shown for various downy mildew groups. Similar transitional phenotypic and ecological characters between the hemibiotrophic or necrotrophic Phytophthoras and the obligate biotrophic downy mildews have been reported in other species and genera [1,40,41]. This is the case with *Phytophthora litchii*, the taxonomic position of which has long been controversial due to the differentiation of *Peronospora*-like sporangiophores [42,43], and all the unculturable obligate biotrophic *Phytophthora* species, including *P. cyperi*, *P. leersiae*, *P. lepironiae*, and *P. polygoni* [4,44,45]. Other examples include the genera *Viennotia*, *Poakatesthia*, and *Sclerophthora*, which possess similar features to *Phytophthora* species, although they are placed among the downy mildews [46,47,48]. In 1952, Gäumann [49] had proposed that *Phytophthora* spp. and downy mildews were likely to be related, but their taxonomic relationship has long been a matter of debate [50,51]. The first phylogenetic studies using nuclear ITS rDNA suggested that the two taxonomic groups potentially form a monophyletic clade [22,52]. This hypothesis was corroborated by subsequent multigene phylogenetic and phylogenomic studies, with all downy mildews unambiguously residing within the genus *Phytophthora* [4,40,41,53,54,55]. Similar results were obtained in the phylogenetic analyses of the present study using a concatenated nuclear and mitochondrial 4-locus dataset. Downy mildews were nested within the *Phytophthora* clades. As shown before by Bourret et al. [4], in addition to *Phytophthora* clades 1–12 and 14, two further clades were identified, one accommodating the DMCC, GDM, and BDM and another one the DMPH. Clade 13 currently consists exclusively of the undescribed *Phytophthora* taxon mugwort [4]. Apparently, in the genus *Phytophthora*, an obligate biotrophic lifestyle evolved independently several times since the DMCC, GDM, and BDM share a common ancestor with *Phytophthora* clades 1–5, 12, and 14, whereas the DMPH, together with the obligate biotrophic *P. cyperi* from clade 14, share a common ancestor with *Phytophthora* clade 1. No close phylogenetic relationship was found between *P. heterospora* and any of the downy mildews included in the analyses, although in previous phylogenetic studies using only single genes, clade 4 species were several times shown to be the nearest relatives to the downy mildews [4,22,43]. However, as reported for the downy mildew-like species *P. litchii* (also clade 4), *P. heterospora* may share some genomic characteristics with the downy mildews forced by environmental and host adaptation during its evolution [43].

Phylogenetically, *P. heterospora* is closely related to *P. palmivora*, from which it can be distinguished by 3–4 fixed polymorphisms in ITS sequences and a total of 18–25 bp differences across the *Btub*, *cox*1, and *nadh*1 gene regions. Nonetheless, due to the high intraspecific variability ([18], this study) and the lack of any type material or key specimens for *P. palmivora* [56,57] that could serve as DNA sources for robust molecular identification and phylogenetic studies, isolates belonging to *P. heterospora* have often been misidentified in the past. A search of ITS sequences in the National Center for Biotechnology Information (NCBI) database and previous publications revealed that isolates with sequences identical to *P. heterospora* have been recorded from almost all continents and different hosts. The crown and root rot of pomegranate reported in young plantations in Italy and Turkey appear to be most likely caused by *P. heterospora* [58,59]. The lower maximum temperature (<35 °C) for mycelium growth of Turkish isolates and their phylogenetic position indicate that they did not belong to *P. palmivora* [59]. Similarly, the dieback and mortality of young olive trees in a nursery and in plantations in Souk El Arbaa (Morocco) attributed to *P. palmivora* [60] were presumably also caused by *P. heterospora*, as demonstrated by the occurrence of subglobose and non-papillate sporangia, which resemble pseudoconidia. Unfortunately, the identification of the isolates was based exclusively on morphological characteristics, and no DNA sequences are available [60]. All these records, together with the finding of *P. heterospora* from olive plantations in this study, suggest that the pathogen is well-established in agricultural tree crops in areas with Mediterranean and semi-arid climatic conditions, most likely favored by the intensive cultivation systems [61,62]. The clonal nature and high virulence of *P. heterospora*, as well as its restricted distribution to nurseries and new plantings, indicate that it is an emerging and potentially invasive pathogen recently introduced via infected plants [63,64]. This hypothesis is supported by the abundant detection of *P. heterospora* in asymptomatic potted plants of ten different hosts in two large European retail nurseries using a qPCR technique [65].

The current distribution of *P. heterospora* in the field, however, may be limited by hot temperatures and drought in summer, which are not favorable to the requirements of the pathogen for growth and sporulation. Similar to its closest relative, *P. palmivora*, *P. heterospora* might also be more adapted to an aerial lifestyle, requiring high humidity [39,66], as revealed by the findings from durian trees in tropical Southeast Asia, including Indonesia, Malaysia, Thailand, and Vietnam (this study). The morphological and physiological attributes of *P. heterospora* support its ability to behave either as an aerial or soilborne pathogen. The production of caducous pseudoconidia, in addition to the caducous sporangia, provides *P. heterospora* with an ecological advantage in durian farms in southern Vietnam, as during monsoon season pseudoconidia are spread aerially by rain and wind and may infect stems and branches. During the dry season, water splashes caused by sprinkler irrigation systems may also favor aerial infections [39,66]. The ability to cause cankers on fruit-bearing trees of durian suggests that *P. heterospora* may be an aggressive pathogen, provided that environmental conditions are conducive, i.e., in tropical and subtropical regions. In addition, some key morphological properties, such as a heterothallic breeding system, an oospore wall index of only 0.33, caducity of pseudoconidia and sporangia, and thick-walled chlamydospores, indicate a potential center of origin of *P. heterospora* in tropical regions, most likely Southeast Asia, Central America, or West Africa, as suggested for most clade 4 species [67,68]. The finding of an isolate with identical ITS sequence to *P. heterospora* (GenBank accession: KY475630) from *T. cacao* in Ghana, a neighbor country to the Ivory Coast, where *P. palmivora* var. *heterocystica* was originally described, supports the hypothesis of a West African origin [1,14]. However, to ascertain the evolutionary and geographic origin of *P. heterospora*, further studies are needed, including surveys in yet non-surveyed natural environments to detect the pathogen on co-evolved hosts without causing diseases [69,70], followed by phenotyping and genotyping studies using a global population [71,72,73].

The differences in the proportion of pseudoconidia and sporangia production recorded (1) for the same *P. heterospora* isolates between solid dry agar and water culture and (2) between *P. heterospora* isolates from Mediterranean regions in Italy and humid tropical Vietnam are of high interest. The ability of *P. heterospora* to partially switch between asexual structures depending on the substrate of growth, i.e., increase in pseudoconidia production on solid agar and sporangia production in water, respectively, indicates that this trait evolved as an adaptation to specific environmental conditions [18]. Therefore, isolates of *P. heterospora* from Mediterranean and semi-arid regions may be on the evolutionary path towards a lifestyle as a conidial plant pathogen with reduced dependence on free water, while isolates from Vietnam apparently prefer sporangia due to the conducive conditions in humid tropical environments [39]. Spore formation and germination involve the staged expression of a large subset of the transcriptome, commensurate with the importance of spores in the life cycle. Therefore, further studies using new technologies such as RNA-seq may be effective in understanding spore biology and the pathogenic mechanisms of *P. heterospora* [74].

Remarkably, isolates PH083 and PH090 from *A. unedo* in Italy belonged to a discreet lineage in an intermediate evolutionary position between *P. heterospora* and *P. palmivora* and, consequently, were designated as *P.* taxon *palmivora*-like. Both nuclear gene sequences and the absence of pseudoconidia characterize these isolates as *P. palmivora*. However, their *cox*1 and *nadh*1 sequences are identical to *P. heterospora*, i.e., they share the same maternal line. This feature was previously reported for the three hybrid species in clade 7a—namely, *P.* × *cambivora*, *P.* × *heterohybrida*, and *P.* × *incrassata*, which share the same *nadh*1 genotype and, hence, most likely the same maternal parent [75]. Interestingly, the occurrence of multiple heterozygous sites in the *Btub* sequences of both *P.* taxon *palmivora*-like (7) and *P. palmivora* (10) suggests that they may originate from sexual hybridization rather than somatic fusion, a feature common to all known *Phytophthora* hybrids [76,77]. However, further molecular analyses, such as cloning, sequencing of other nuclear and mitochondrial genes, and estimation of nuclear DNA content and ploidy level by flow cytometry, are required to confirm this hypothesis [75].

Our finding of high phenotypic and genetic variability amongst *P. palmivora* isolates is in accordance with previous studies [18,67,78]. The two isolates from jackfruit in Vietnam [21] were genetically different from all the other *P. palmivora* isolates. Interestingly, they originate from the same area of the Mekong River Delta as the *P. heterospora* isolates from durian trees [21]. Further investigations are urgently needed to characterize the global population structure of *P. palmivora*, considering its panglobal distribution and high impact on a wide range of horticultural and ornamental plant species, as well as on natural wild ecosystems [39].

## 5. Conclusions

Our study demonstrates that the *Phytophthora* isolates from olive trees in Italy, characterized by the unique ability to produce pseudoconidia, belong to a new species described here as *P. heterospora* sp. nov. Italy is one of the leading olive producers, with more than one million hectares, accounting for about 10% of the world’s cultivation area [79]. The severity of symptoms observed on infected trees, together with the results from pathogenicity tests, indicate that the pathogen could represent a new emerging threat to olive cultivation, particularly in newly established plantations, where pathogen infection appears to be favored by agronomic practices and recurrent water irrigation. Over the last few years, an increasing number of *Phytophthora* species have been associated with a decline in olive trees in Mediterranean regions [80,81,82,83,84].

Likewise, the impact of *P. heterospora* on durian production in Vietnam may be underestimated. The durian industry in Vietnam is small but rapidly expanding, catering mainly for the domestic market, with some export trade with Taiwan [39]. In many cases *P. heterospora* may have been misidentified as *P. palmivora*, and previous reports of *P. palmivora* as a pathogen of durian in Vietnam should be reconsidered if they were not supported by phylogenetic analysis.

Finally, nurseries provide an environment where *P. heterospora* is widespread in Italy, Iran, and Vietnam, mainly as a soilborne pathogen infecting fine roots of several hosts, including agricultural tree crops and forest species. This highlights, once again, the primary role of nurseries in spreading *Phytophthora* pathogens with uncertified plant material into new ecosystems [63,64].

## Figures and Tables

**Table 1:** Details of *Phytophthora* isolates used in the morphological, growth-temperature, and pathogenicity studies.

*Phytophthora* Taxa	Isolate Codes ^1^	Country, Region	Year	Host
*P. heterospora*	PH047	Italy, Sardinia	2010	*Olea europaea*
*P. heterospora*	PH051 (CBS 148035)	Italy, Sardinia	2010	*Olea europaea*
*P. heterospora*	PH052	Italy, Sardinia	2010	*Olea europaea*
*P. heterospora*	PH054 (CBS 148034) (T)	Italy, Sardinia	2010	*Olea europaea*
*P. heterospora*	PH057	Italy, Sardinia	2010	*Olea europaea*
*P. heterospora*	PH211	Italy, Sardinia	2013	*Juniperus oxycedrus*
*P. heterospora*	317-A12 (CBS 148036)	Italy, Sicily	2014	*Capparis spinosa*
*P. heterospora*	Palm2 ^2^	Italy, Calabria	1999	*Olea europaea*
*P. heterospora*	Campobello 2b	Italy, Sicily	2005	*Olea europaea*
*P. heterospora*	DB2	Vietnam, Mekong Delta	2013	*Durio zibethinus*
*P. heterospora*	A1A	Vietnam, Mekong Delta	2013	*Durio zibethinus*
*P. heterospora*	A1B1	Vietnam, Mekong Delta	2013	*Durio zibethinus*
*P. heterospora*	C2B1	Vietnam, Mekong Delta	2013	*Durio zibethinus*
*P. palmivora*	CBS 179.26 ^3^	Sri Lanka, n.a.	1979	*Theobroma cacao*
*P. palmivora*	Phoenix4 ^4^	Italy, Sicily	2005	*Phoenix canariensis*
*P. palmivora*	PhoenixF ^4^	Italy, Sicily	2005	*Phoenix canariensis*
*P. palmivora*	GRE1 (IMI 390579) ^5^	Italy, Sicily	2002	*Grevillea rosmarinifolia*
*P. palmivora*	MD5 (IMI 503890) ^6^	Vietnam, Mekong Delta	2013	*Artocarpus heterophyllus*
*P. palmivora*	MD6 (IMI 503891) ^6^	Vietnam, Mekong Delta	2013	*Artocarpus heterophyllus*
*P*. taxon *palmivora*-like	PH083	Italy, Sardinia	2011	*Arbutus unedo*
*P*. taxon *palmivora*-like	PH090	Italy, Sardinia	2011	*Arbutus unedo*

^1^ Abbreviations of isolates and culture collections: CBS = Centraalbureau voor Schimmelcultures, Utrecht, Netherlands; IMI = CABI Bioscience, United Kingdom; other isolate names and numbers are as given by the collectors; T = ex-type culture. ^2^ Isolate from Cacciola et al. [12]. ^3^ Isolate from Brasier and Griffin [18]. ^4^ Isolates from Pane et al. [19]. ^5^ Isolate from Cacciola et al. [20]. ^6^ Isolates from Van Tri et al. [21].

**Table 2:** Proportion (%) of pseudoconidia, sporangia, and chlamydospores produced by *Phytophthora heterospora*, *P.* taxon *palmivora*-like and *P. palmivora* at 20 °C on solid carrot agar (CA) and on CA submerged in nonsterile soil extract water.

*Phytophthora* spp.	Pseudoconidia/Sporangia/Chlamydospores	
	CA	Water
*P. heterospora* ^1^	86/8/6	74/24/2
*P. heterospora* ^2^	34/56/10	26/74/8
*P. taxon palmivora*-like	-/82/18	-/85/15
*P. palmivora*	-/78/22	-/80/20

^1^ Isolates from *Capparis spinosa*, *Juniperus oxycedrus*, and *Olea europaea* in Italy. ^2^ Isolates from *Durio zibethinus* in Vietnam.

## Data Availability

All sequences generated during this study are available from GenBank and accession numbers are given in Table S1. All datasets and trees derived from BI and ML analyses are available from DRYAD (https://datadryad.org) (Dryad Dataset, https://doi.org/10.5061/dryad.1jwstqjvx).

## Supplementary Materials

The following are available online at https://www.mdpi.com/article/10.3390/jof7100870/s1, Table S1: Information of all oomycete isolates used in the phylogenetic analyses, including local, international, and alternative isolate identifications, Table S2: Base pairs differences across *Btub*, ITS, *Cox*1, *nadh*1 sequences showing inter- and intraspecific variation of *Phytophthora heterospora*, *P. palmivora*, and *P.* taxon *palmivora*-like.
