# Supplementary material for: Phytophthora heterospora sp. nov., a New Pseudoconidia-Producing Sister Species of P. palmivora
Source: J Fungi (Basel). 2021 Oct 16;7(10):870. doi: 10.3390/jof7100870 (PMC8539753; doi:10.3390/jof7100870)
Supplement: Supplementary file 1 [file jof-07-00870-s001.zip › Supplementary files/Table S1.pdf]

Table S1. Information of all oomycete isolates used in the phylogenetic analyses, including local, international and alternative isolate identifications. GenBank accession numbers for sequences obtained in the present study are printed in bold.

(Sub) Clade	Species	Isolate identification	GenBank accession No.			
			ITS	*Btub*	*cox*1	*nadh*1
-	*Nothophytophthora valdiviana* (T)	CBS 142357; CL331	MZ753910	MZ736449	MZ736421	KY788628
1	*Phytophthora nicotianae* (ASI2)	WPC P6303; CPHST BL 162	(GS) JAAKBE010001860	(GS) JAAKBE010003943	(GS) JAAKBE010002284	(GS) JAAKBE010003823
1a	*P. cactorum*	P295	(GS) RCMO01001417	(GS) RCMO01000030	(GS) RCMO01000985	(GS) RCMO01000778
1c	*P. infestans*	CBS 147289, T30-4; TJ1504	MZ753914	MZ736454	MZ736428	**MZ736507**
2a	*P. meadii*	CBS 219.88; 61J9; IMI 129185; WPC P3950 - P3433; ATCC 58103	MH620109	KX250594	Contig HQ708324 MT583652	MT583705
2b	*P. tropicalis* (T)	CBS 434.91; WPC P10329; 35C8; ATCC 76651, MYA-4218; CPHST BL 58	MH620112	KX250699	Contig MH620025 MH136987	-
2b	*P. tropicalis*	AN97/86; PD97/11132	-	-	-	AY563988
2c	*P. multivora* (T)	CBS 124094; 55C5; WPC P16837, P19594; WAC13201; ICMP 19454	MG865546	KX250776	Contig MH136939 FJ237508	**MZ736508**
2e	*P. multivesiculata* (T)	CBS 545.96; 29E3; WPC P10410; CPHST BL 50G; MG 33-6; AR 239; PD 95/8679	MH620118	EU080066	Contig MH136937 MH620032	KX011295
3	*P. nemorosa* (T)	CBS 114870; WPC P19600; 41C4; CPHST BL 27; ATCC MYA-2948	KF317082	KX250965	Contig KF317104 MH136941	DQ361211
3	*P. pseudosyringae* (T)	CBS 111772; WPC P10437; 30A8; CPHST BL 51G; ATCC MYA-4222; p284; PSEU 6	KF317083	KX250979	**MZ736504**	**MZ736509**
4	*P. alticola*	CBS 121939; WPC P16948 - P16052; CMW 34279 - 19425; 47G5	KF317084	KX251007	Contig HQ261245 KF317106	-
4	*P. arenaria* (T)	CBS 127950; WPC P19599; PD 825; 55C2; CPHST BL 78	HQ013219	KJ372289	Contig MH136848 MH620034	-

(Sub) Clade	Species	Isolate identification	GenBank accession No.			
			ITS	*Btub*	*cox*1	*nadh*1
4	*P. boodjera* (T)	CBS 138637; VHS 26806; CPHST BL 181; WPC 13637	KJ372244	KJ372283	Contig<br>KJ396688<br>MH477743	-
4	*P. cathayensis* (T)	CP30; CGMCC 19655	MN385741	MT063102	MN692211	-
4	*Phytophthora heterospora*	317-A12; CBS 148036	**MT232395**	**MZ782810**	**MZ782831**	**MZ782852**
4	*P. heterospora*	A1A	**MT232399**	**MZ782813**	**MZ782834**	**MZ782855**
4	*P. heterospora*	A1B1	**MZ927098**	**MZ782814**	**MZ782835**	**MZ782856**
4	*P. heterospora*	C2B1	**MZ927099**	**MZ782815**	**MZ782836**	**MZ782857**
4	*P. heterospora*	DB2	**MZ927100**	**MZ782816**	**MZ782837**	**MZ782858**
4	*P. heterospora*	FK1	**KM979514**	**KU870332**	**KU870322**	**KP050547**
4	*P. heterospora*	FK2	**KP050543**	**KU870333**	**KU870323**	**KP050548**
4	*P. heterospora*	FK3	**KP050544**	**KU870334**	**KU870324**	**KP050549**
4	*P. heterospora*	FK4	**KP050545**	**KU870335**	**KU870325**	**KP050550**
4	*P. heterospora*	FK5	**KP050546**	**KU870336**	**KU870326**	**KP050551**
4	*P. heterospora*	Campobello2b	**MT232397**	**MZ782812**	**MZ782833**	**MZ782854**
4	*P. heterospora*	Palm2	**MT232396**	**MZ782811**	**MZ782832**	**MZ782853**
4	*P. heterospora*	PH047	**MZ927095**	**MZ782804**	**MZ782825**	**MZ782846**
4	*P. heterospora*	PH051; TJ0237; CBS 148035	**MT232392**	**MZ782805**	**MZ782826**	**MZ782847**
4	*P. heterospora*	PH052	**MZ927096**	**MZ782806**	**MZ782827**	**MZ782848**
4	*P. heterospora* (T)	PH054; TJ0238; CBS 148034	**MT232393**	**MZ782807**	**MZ782828**	**MZ782849**
4	*P. heterospora*	PH057	**MZ927097**	**MZ782808**	**MZ782829**	**MZ782850**
4	*P. heterospora*	PH211	**MT232394**	**MZ782809**	**MZ782830**	**MZ782851**
4	*P. litchii* (ASI1)	CBS 100.81; WPC P15218; CPHST BL 48G; ATCC 34595; MG 33-3	MG865525	MH493966	MH136920	DQ361208
4	*P. litchii*	SHS3	-	(GS)<br>PCFV01000014	(GS)<br>PCFV01000263	(GS)<br>PCFV01000263
4	*P. megakarya* (T)(A1)	IMI 182590; WPC 1664; CPHST BL 22	MG865533	-	Contig<br>MH136928<br>AY564193	-
4	*P. megakarya*	IMI 337098	-	AY564078	-	AY564020
4	*P. megakarya* (T)(A2)	CBS 238.83; 61J5; WPC P1672; CPHST BL 73; ATCC 42100; IMI 202077	MG865534	KX251035	Contig<br>MH136929<br>MH620035	-

(Sub) Clade	Species	Isolate identification	GenBank accession No.			
			ITS	*Btub*	*cox*1	*nadh*1
4	*P. megakarya*	zdho	-	-	-	(GS) NBNE01000504
4	*P. palmivora*	CBS 179.26; IMI 198927; WPC P3548	**MT232400**	**MZ782817**	**MZ782838**	**MZ782859**
4	*P. palmivora*	Phoenix4	**MT232403**	**MZ782819**	**MZ782840**	**MZ782861**
4	*P. palmivora*	PhoenixF	**MT232402**	**MZ782818**	**MZ782839**	**MZ782860**
4	*P. palmivora*	IMI 390579; GRE1	**MT232404**	**MZ782820**	**MZ782841**	**MZ782862**
4	*P. palmivora*	IMI 503890; MD5	**MT232406**	**MZ782821**	**MZ782842**	**MZ782863**
4	*P. palmivora*	IMI 503891; MD6	**MT232407**	**MZ782822**	**MZ782843**	**MZ782864**
4	*P.* taxon *palmivora*-like	PH083	**MZ927101**	**MZ782823**	**MZ782844**	**MZ782865**
4	*P.* taxon *palmivora*-like	PH090	**MT232401**	**MZ782824**	**MZ782845**	**MZ782866**
4	*P. palmivora* (ASI1)	CBS 305.62; WPC P0633; CPHST BL 105; IMI 062655; IMI 348384	MG865559	MH493992	MH136949	-
4	*P. palmivora* (ASI2)	WPC P0255; CPHST BL 46; ATCC 26200	MG865560	MH493994	MH136950	-
4	*P. palmivora*	ZC01	(GS) VOQZ01000473	(GS) VOQZ0100	(GS) VOQZ01000950	(GS) VOQZ01000945
4	*P. quercetorum* (T)	CBS 121119; WPC P15555; ATCC MYA-4086; CPHST-BL 52G; MD 9/2	MG865577	EU080901	Contig MH136969 KX759520	-
5	*P. castaneae*	CBS 587.85; WPC 15598; 61J7; IMI 325914; ATCC 36818; ICMP 19450	MH620122	KX251098	(MGS) MN883602	(MGS) MN883602
5	*P. heveae* (T)	CBS 296.29; WPC P3428; 22J1; ATCC 58815; ICMP 19451; KACC 44943; IMI 180616; CPHST BL 67	MH620123	KX251112	(MGS) MN883604	(MGS) MN883604
6a	*P. humicola* (T)	CBS 200.81; WPC P3826; 32F8; ATCC 52179, MYA-4080; IMI 302303; p198; CPHST BL 42G;	KF112855	KX251140	Contig KF112862 HQ708305	**MZ736506**
6b	*P. chlamydospora*	P17-99	(GS) JAABLK010000522	(GS) JAABLK010000021	(MGS) CM022726	(MGS) CM022726
7a1	*P. tyrrhenica* (T)	CBS 142301; PH154; TJ1153	KU899188	KU899265	MZ736441	KU899507
7a2	*P. europaea* (T)	CBS 109049; 62A2; WPC P10324; EUR 2; CPHST BL 37G	MH620138	KX251523	Contig KU681022 HQ261303	KU899469
7b	*P. sojae*	28F9; P6497	KC733444	KX251770	(MGS) DQ832717	(MGS) DQ832717

(Sub) Clade	Species	Isolate identification	GenBank accession No.			
			ITS	*Btub*	*cox*1	*nadh*1
7c	*P. cinnamomi* (T)	CBS 144.22; IMI 022938; 61J1; WPC P2110; ATCC 1407	KU899160	KX251812	Contig HQ708257 MH620070	KU899475
8a	*P. sansomeana*	WPC P8051; 47H3; ATCC MYA-4455; CPHST BL 55	MH620155	KX251931	(MGS) MH936679	(MGS) MH936679
8b	*P. lactucae* (T)	61F4; BPIC 1985; P19872; CPHST BL 113	MH620161	KX252043	Contig MH620085 KC478738	KF882714
8c	*P. ramorum* (T)	CBS 101553; P1577; BBA9/95; Pr-164; CSL2266; WPC P10103; PR-05-012; CPHST BL 55G	MG865581	(GS) RYEP01000230	(MGS) EU427470	(MGS) EU427470
9a2	*P. quininea* (T)	CBS 407.48; 46C4; ATCC 46733; WPC P1089-P8488; CPHST BL 54G	MG865580	AY564085	Contig AY564200 HQ708386	AY564027
9b1	*P. captiosa* (T)	CBS 119107; 46H7; CPHST BL 11; NZFS 310C; WPC P10719; ICMP 15576	DQ297402	-	-	-
9b2	*P. captiosa*	46H8; NZFS 430; ICMP 17567	-	KX252555	(MGS) MN883606	(MGS) MN883606
10	*P. boehmeriae* (T)	CBS 291.29; WPC P6950; IMI 180614; MG 42-6; 45F9; CPHST BL 32G; ATCC 60238	HQ643149	EU080162	Contig HQ261251 KT183047	DQ361200
10	*P. kernoviae* (T)	WPC P19827; IMI 393170; CPHST BL 91; PCPCC P1571	(GS) VKKV01000063	(GS) VKKV01000263	(GS) VKKV01000418	(GS) VKKV01000418
11	*P. lilii* (T)	CBS 135746; NBRC 32174; MAFF 237500; EL 8701; CPHST BL 123	MG865523	Contig MG493965 AB856782	MH136918	-
12	*P. castanetorum* (T)	CBS 142299; BD292	MF036182	MZ736453	MZ736427	MF036292
12	*P. quercina*	TJ292; PL7; P731	MF036194	**MZ736504**	**MZ736505**	MF036301
12	*P. tubulina* (T)	CBS 141212; TJ271; TUB1; Hohenegg II/1; P736	MF036196	MZ736467	MZ736440	MF036303
14	*P. cyperi*	11048	MK045313	-	-	-
14	*P. cyperi*	MVAP06098582	-	KY608800	MH445454	.
DMPH	*Bremia lactucae*	SF5	(GS) SHOA01000013	(GS) SHOA01000026	(MGS) MH271689	(MGS) MH271689

(Sub) Clade	Species	Isolate identification	GenBank accession No.			
			**ITS**	***Btub***	***cox*1**	***nadh*1**
DMPH	*Plasmopara halstedii*	Plhal710	(GS) PDFJ01000023	(GS) PDFJ01000039	(GS) PDFJ01000150	(GS) PDFJ01000150
DMPH	*Plasmopara viticola*	INRA-PV221	(GS) MBPM02000295	(GS) MBPM02000031	(MGS) MN105125	(MGS) MN105125
DMCC	*Peronospora effusa*	SPp3	MG793197	-	-	-
DMCC	*Peronospora effusa*	R14	-	(GS) QLLG01000479	(MGS) MH325167	(MGS) MH325167
DMCC	*Peronospora tabacina*	968-S26	(GS) NBSG01000065	(GS) NBSG01000797	(MGS) KT893456	(MGS) KT893456
DMCC	*Pseudoperonospora cubensis*	MSU-1	(GS) AHJF01002487	(GS) AHJF01000065	(GS) AHJF01005719 AHJF01021320	(GS) AHJF01000547
GDM	*Sclerospora graminicola*	UoM-SG	(GS) MIQA02020524	(GS) MIQA02005411	(GS) MIQA02016028	(GS) MIQA02022157
BDM	*Hyaloperonospora arabidopsidis*	Cala2	(GS) LKIA01008204	(GS) LKIA01000455	(GS) LKIA01003057	(GS) LKIA01001610
BDM	*Hyaloperonospora brassicae*	HV2163	EU049248	(GS) SZZJ01000824	(GS) SZZJ01002632	-

(T) Type;
(DMPH) Downy Mildews with Pyriform Haustoria;
(DMCC) Downy Mildews with Colored Conidia;
(GDM) grass-infecting Graminicolous Downy Mildews;
(BDM) Brassicolous Downy Mildews;
(GS) Genome Sequence;
(MGS) Mitochondrial Genome Sequence;
(ASI) Abad Selected isolate (from IDphy: molecular and morphological identification of *Phytophthora* based on the types, https://idtools.org).
