# Supplementary material for: Phytophthora heterospora sp. nov., a New Pseudoconidia-Producing Sister Species of P. palmivora
Source: J Fungi (Basel). 2021 Oct 16;7(10):870. doi: 10.3390/jof7100870 (PMC8539753; doi:10.3390/jof7100870)
Supplement: Supplementary file 1 [file jof-07-00870-s001.zip › Supplementary files/Table S2.pdf]

Table S2. Base pairs (bp) differences across *Btub*, ITS, *cox*1, *nadh*1 sequences showing inter- and intraspecific variation of *Phytophthora heterospora* (HET), *P. palmivora* (PAL) and *P.* taxon *palmivora*-like (LIK).

	**Length of alignment (bp)**	**Differences PAL/HET (bp)**	**Differences PAL/LIK (bp)**	**Differences HET/LIK (bp)**	**Differences within PAL (bp)**	**Differences within HET (bp)**
Btub	918	**11-16**	6-14	7-9	0-16	0-2
**ITS**	788	**3-4**	0-1	3	0-1	0
***cox*1**	1346	**1-2**	2	0	0-2	0
***nadh*1**	796	**6**	0-6	0	0-6	0
**Total**	3848	**21-28**	**8-21**	**10-19**	**0-25**	**0-2**
